# Supplementary material for: Drivers and impacts of sediment deposition in Amazonian floodplains
Source: Nat Commun. 2025 Apr 2;16:3148. doi: 10.1038/s41467-025-57495-1 (PMC11965409; doi:10.1038/s41467-025-57495-1)
Supplement: Supplementary file 1 — Supplementary Information [file 41467_2025_57495_MOESM1_ESM.pdf]

Supplementary information for Drivers and impacts of sediment deposition in Amazonian floodplains

Dongyu Feng[1*], Zeli Tan[1*], Sebastien Pinel[2], Donghui Xu[1], João Henrique Fernandes Amaral[3], Alice César Fassoni-Andrade[4], Marie-Paule Bonnet[5], Gautam Bisht[1]

[1]*Atmospheric, Climate, & Earth Sciences Division, Pacific Northwest National Laboratory, Richland, 99354, Washington, USA*

[2]*Centre of education and research on Mediterranean environments (CEFREM), University of Perpignan Via Domitia (UPVD), Perpignan, 66860, Perpignan, France*

[3]*Earth System Science Program, Faculty of Natural Sciences, Universidad del Rosario, Bogota, 111221, Colombia*

[4]*Instituto de Geociências, Universidade de Brasília, Brasília, 70910, Brazil*

[5]*UMR Espace-DEV, Univ Montpellier, IRD, Montpellier, 34000, Montpellier, France*

*Corresponding author.
E-mail: dongyu.feng@pnnl.gov
E-mail: zeli.tan@pnnl.gov

Content of this file

Figure S1~S18

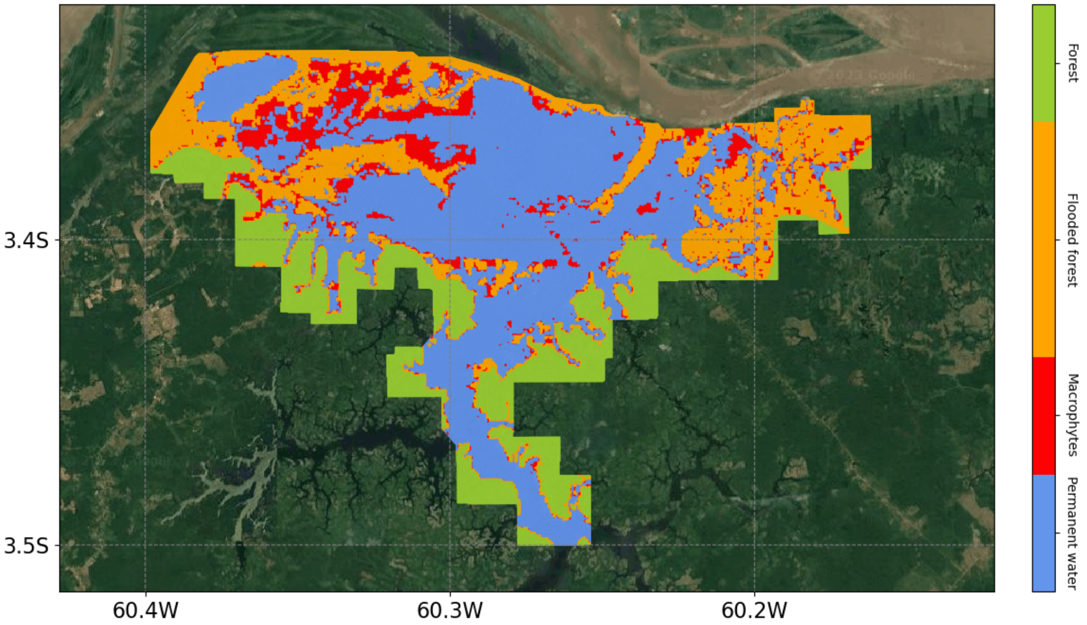

Figure S1 The land cover types in Lake Janauaca: Permanent water, Macrophytes, Flooded forest and Forest. Permanent water consists of Janauaca Lake and river channels. The lake area in Figure 1a is extracted from the permanent water region of the Janauacá floodplain. Macrophytes are aquatic plants growing near water, while forest refers to a large area densely packed with trees and undergrowth on land. Wetlands consist of Macrophytes and Flooded forest. The background map is from Google Satellite Map (Map data ©2024 Google).

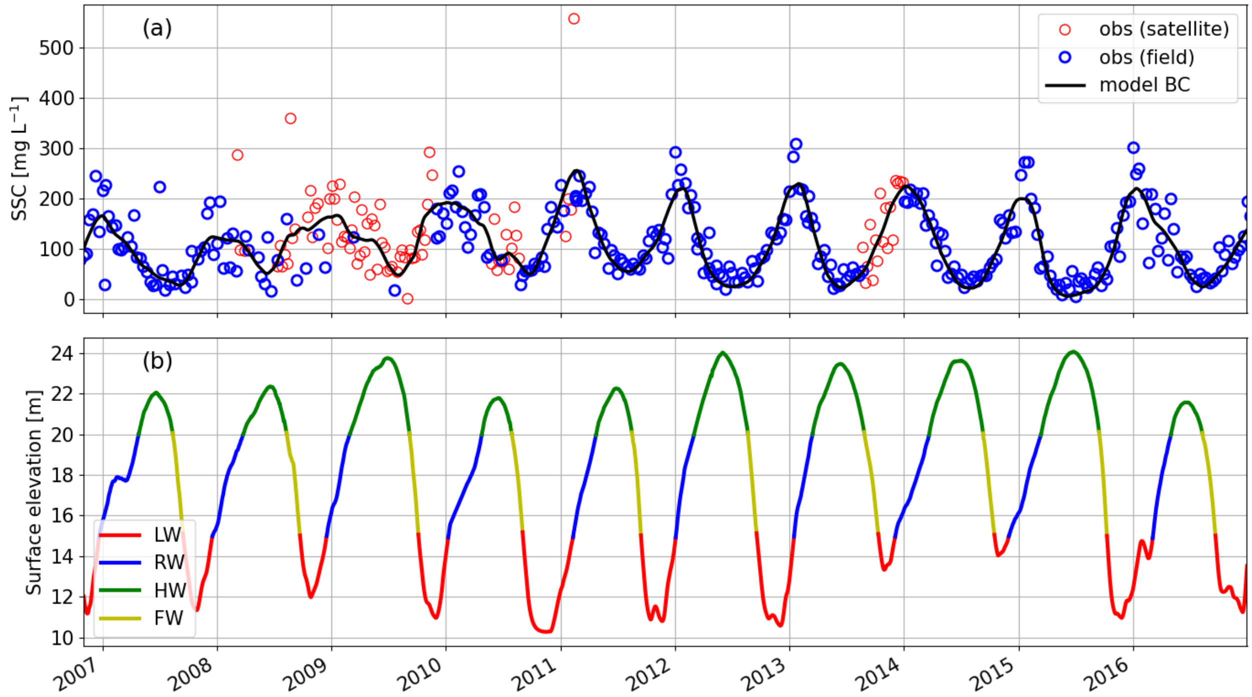

Figure S2 (a) Time series of the SSC data measured at Manacapuru from the observation (blue circle) and from satellite data used to fill the gap periods (red circle) (ORE-HYBAM database, https://hybam.obs-mip.fr/), and the smooth SSC boundary condition used as the model boundary condition after removing outliers and applying the Lowess filter (black line). (b) Time series of water level elevation at water level boundaries (WLBCs) with each hydrological period (LW: low water, RW: rising water, HW: high water, FW: falling water) specified by a different color. The hydrological periods are defined by the water level elevation: LW is when water level < 15 m, HW is when water level > 20 m, and RW and FW are the periods with water level between 15 m and 20 m. The thresholds are chosen because (a) they ensure comparable periods within a hydrological year, and (b) the HW threshold is near the top of the riverbank. Source data are provided as a Source Data file.

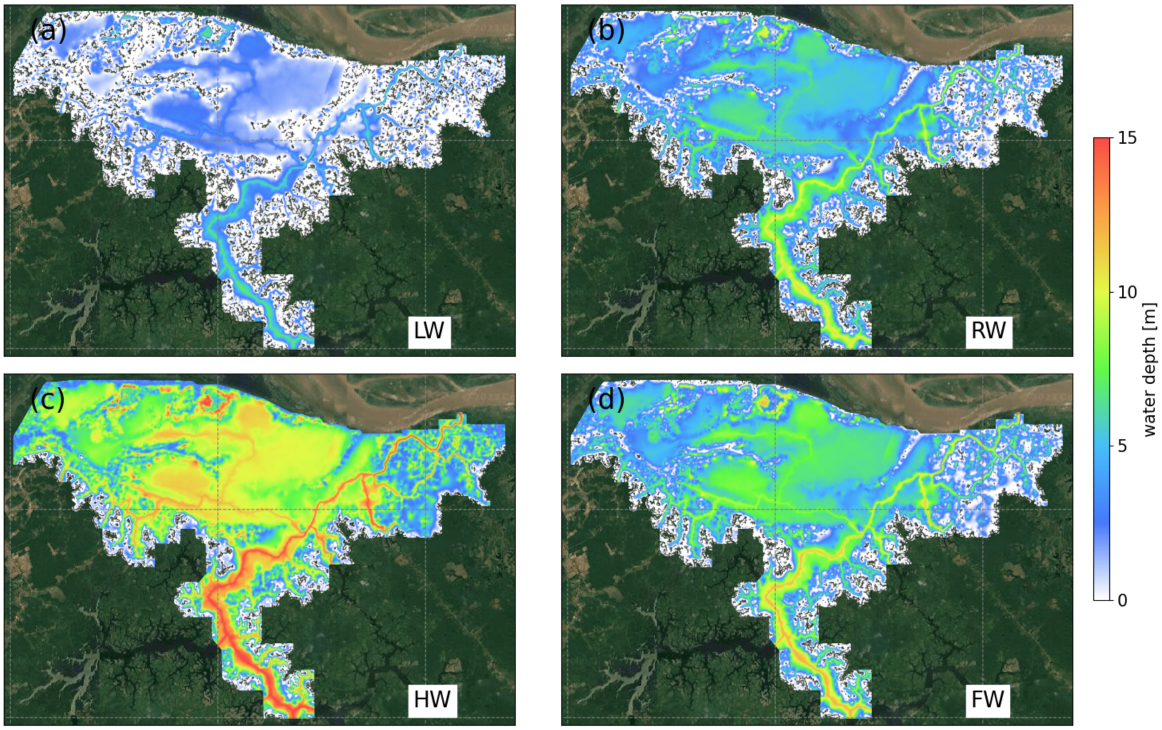

Figure S3 Modeled water depth at representative time instants during the four hydrological periods (LW: Dec 15, 2014 (a), RW: Mar 1, 2015 (b), HW: Jun 15, 2015 (c) and FW: Sep 1, 2015 (d)). The background maps are from Google Satellite Map (Map data ©2024 Google).

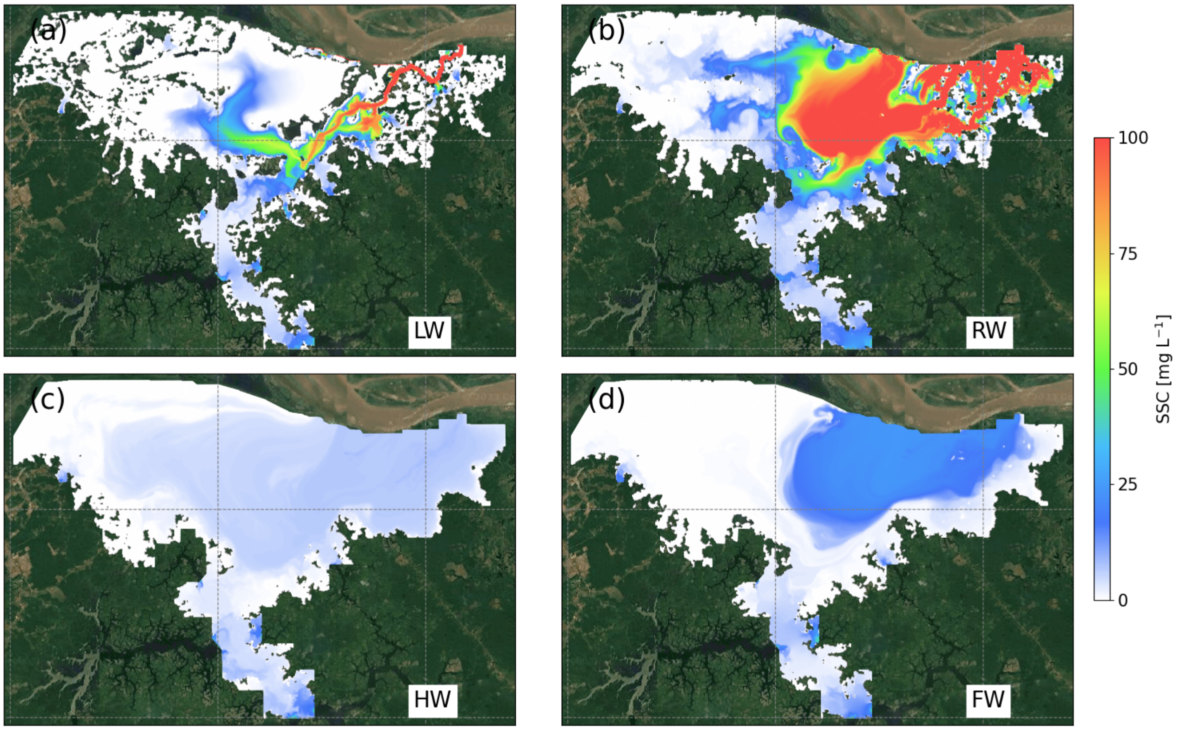

Figure S4 Modeled SSC at representative time instants during the four hydrological periods (LW: Dec 15, 2014 (a), RW: Mar 1, 2015 (b), HW: Jun 15, 2015 (c) and FW: Sep 1, 2015 (d)). The background maps are from Google Satellite Map (Map data ©2024 Google).

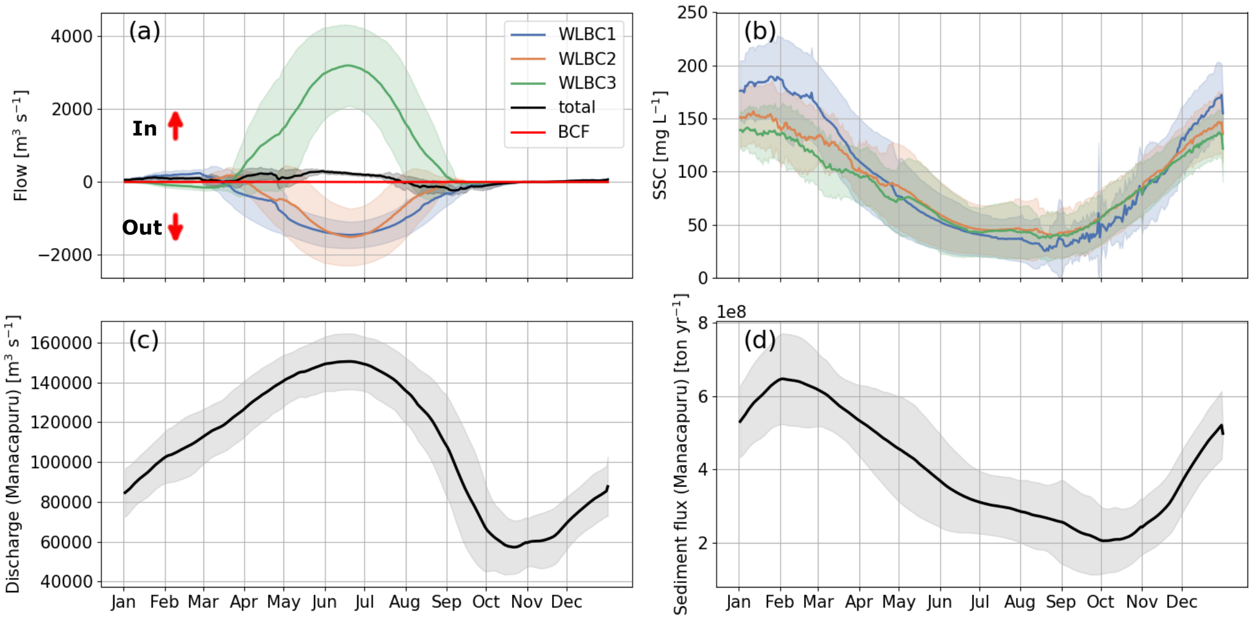

Figure S5 (a) The daily averaged flux of exchanging water flow between Lake Janauacá and the Amazon River mainstem at the three open boundaries WLBC1, WLBC2 and WLBC3 (Fig. 1a), their net total (black) and the total input from 8

upstream inflow (BCF) boundaries (red). (b) The daily averaged SSC at the WLBCs. (c) and (d) are the daily averaged discharge and sediment flux, respectively, at the Manacapuru gauge. The positive and negative values represent the flux into and out of the Janauacá floodplain, respectively. Light shades indicate the variation over multiple simulation years. The channelized flow and overflow are delineated at WLBC1 and WLBC2/WLBC3, respectively, where the bottom elevation at WLBC1 is below 12 m and at WLBC2 and WLBC3, it is over 20 m. From the late LW period to the mid-RW, channelized flow predominantly influences water and sediment exchange at WLBC1, transitioning to overflow in WLBC2 and WLBC3 when the water level exceeds the levee crest. Source data are provided as a Source Data file.

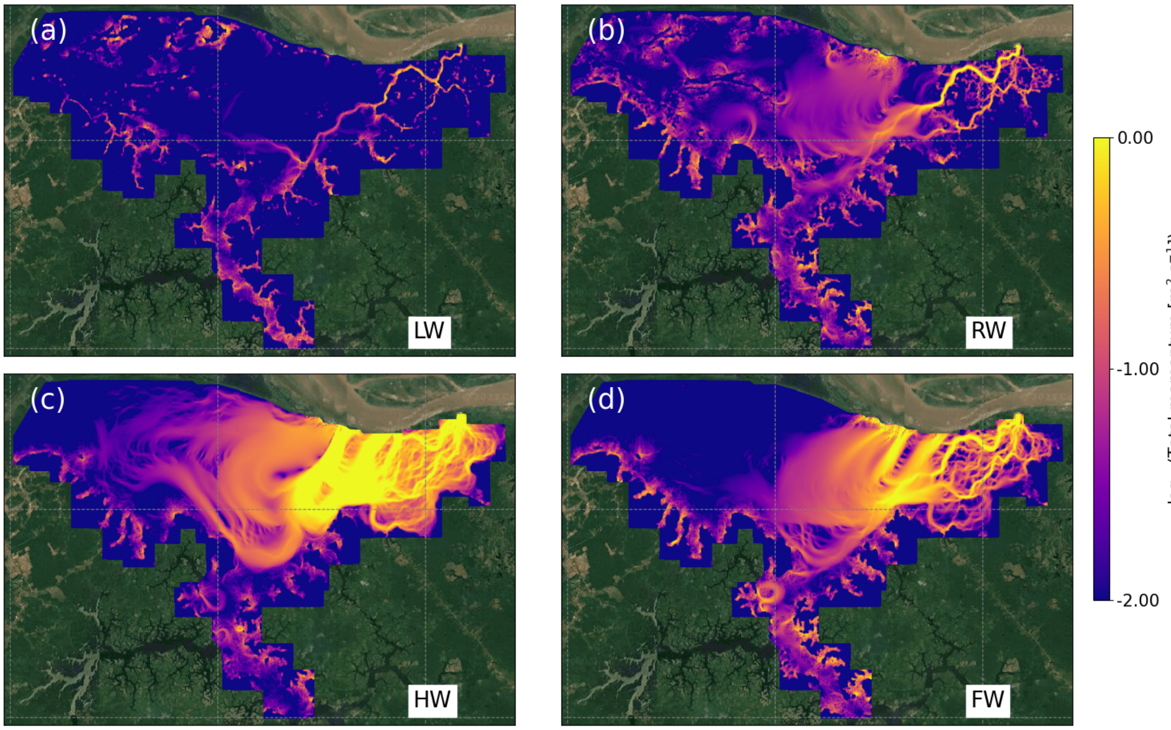

Figure S6 Magnitude of total momentum ($M_{total} = \sqrt{(u \times h)^2 + (v \times h)^2}$) at representative time instants during the four hydrological periods (LW: Dec 15, 2014 (a), RW: Mar 1, 2015 (b), HW: Jun 15, 2015 (c) and FW: Sep 1, 2015 (d)), where h, u and v are water depth, velocities in x and y directions, respectively. The background maps are from Google Satellite Map (Map data ©2024 Google).

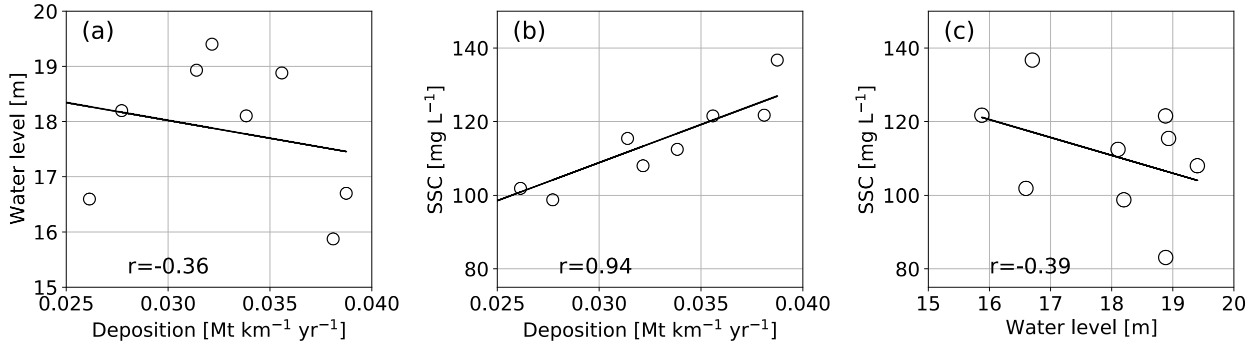

Figure S7 Scatter plots of annual mean deposition rate, water level and SSC: (a) deposition rate vs. water level; (b) deposition rate vs. SSC; (c) water level vs. SSC. The black lines are the best-fit lines. Source data are provided as a

Source Data file.

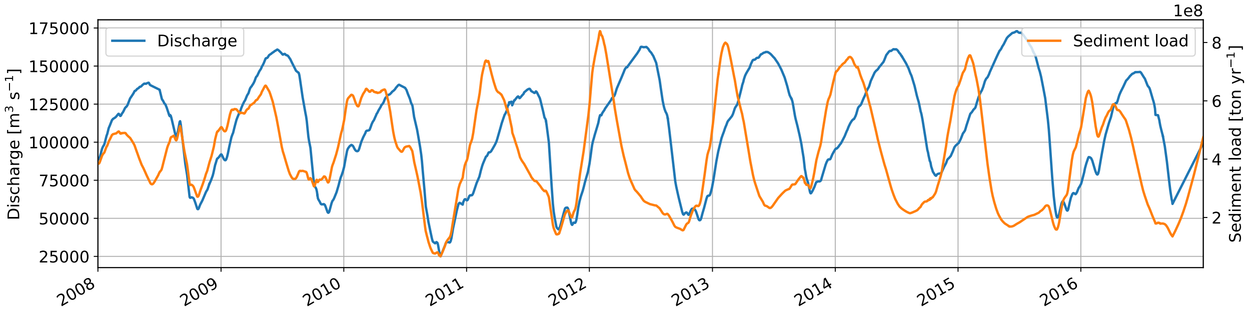

Figure S8 Time series of Amazon River discharge and sediment load measured at Manacapuru. Source data are provided as a Source Data file.

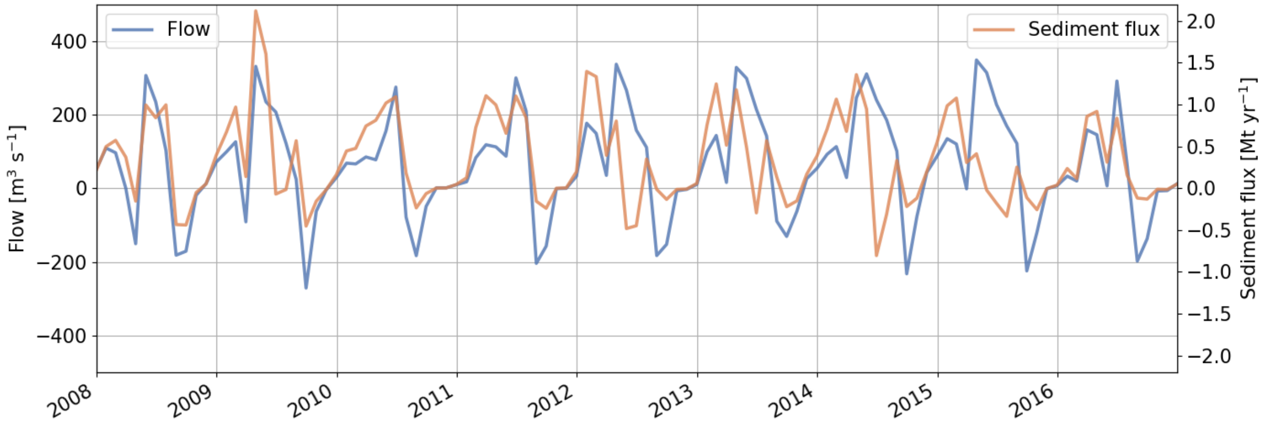

Figure S9 Time series of monthly exchanging rate of flow and suspended sediment through the open boundaries (transects in Figure 1a). The positive and negative values represent the flux into and out of the Janauacá floodplain, respectively. Source data are provided as a Source Data file.

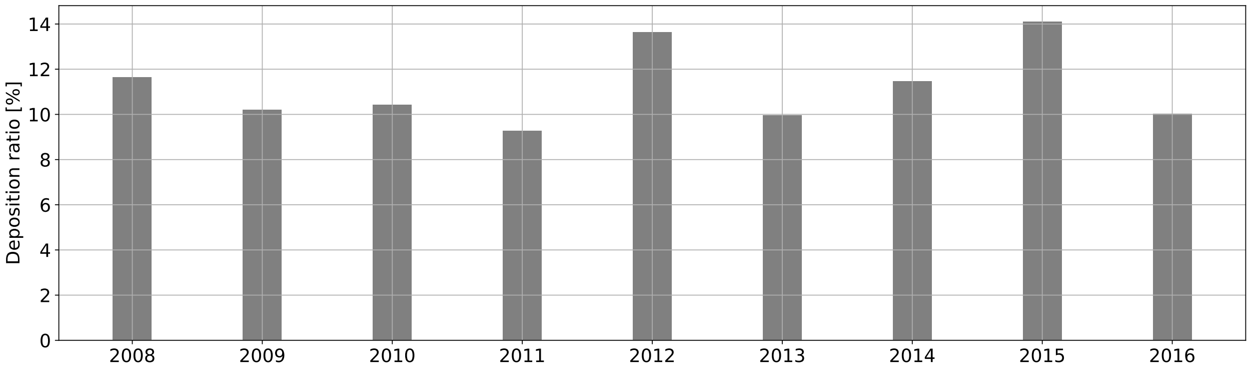

Figure S10 The deposition ratio over the simulation years. The deposition ratio is the proportion of sediment deposited

in the floodplain relative to the sediment influx. Source data are provided as a Source Data file.

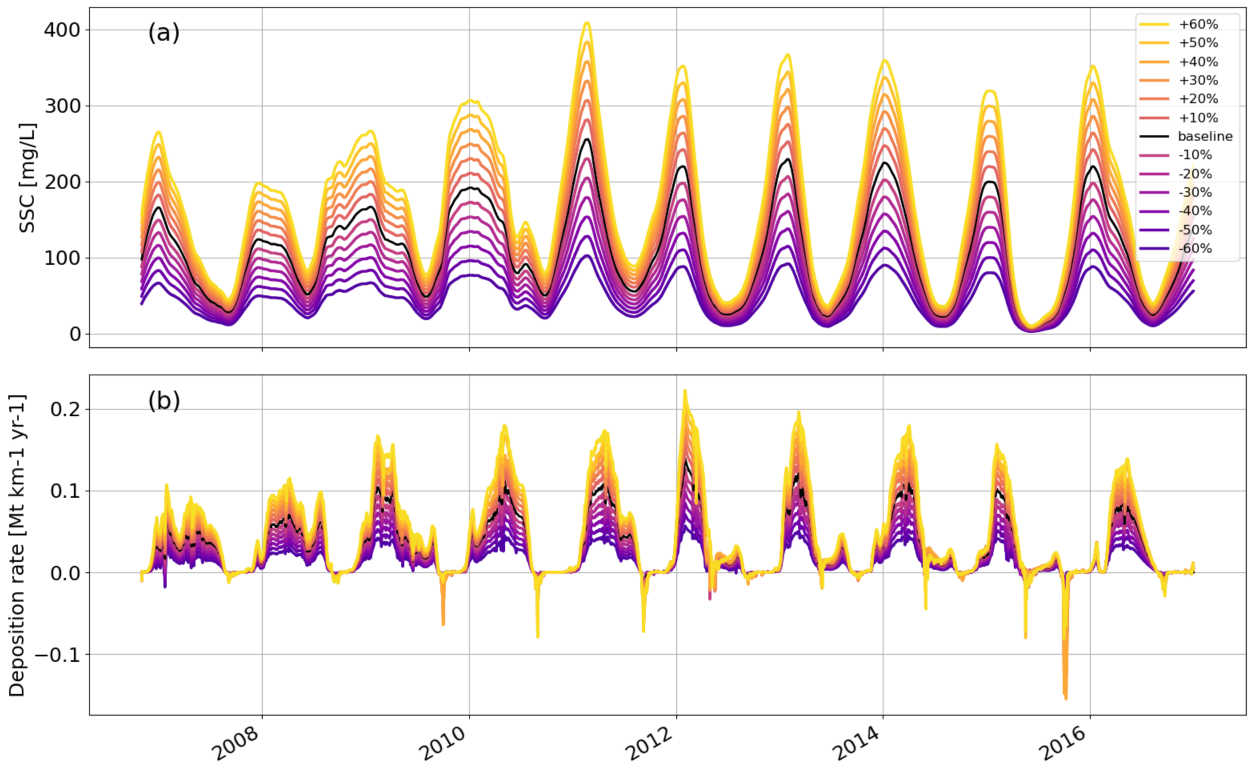

Figure S11 (a) Time series of boundary SSC of the baseline (black) and experiment simulations (colored). (b) The modeled deposition rate of the baseline (black) experiment simulations (colored). Negative deposition rate is due to erosion. Source data are provided as a Source Data file.

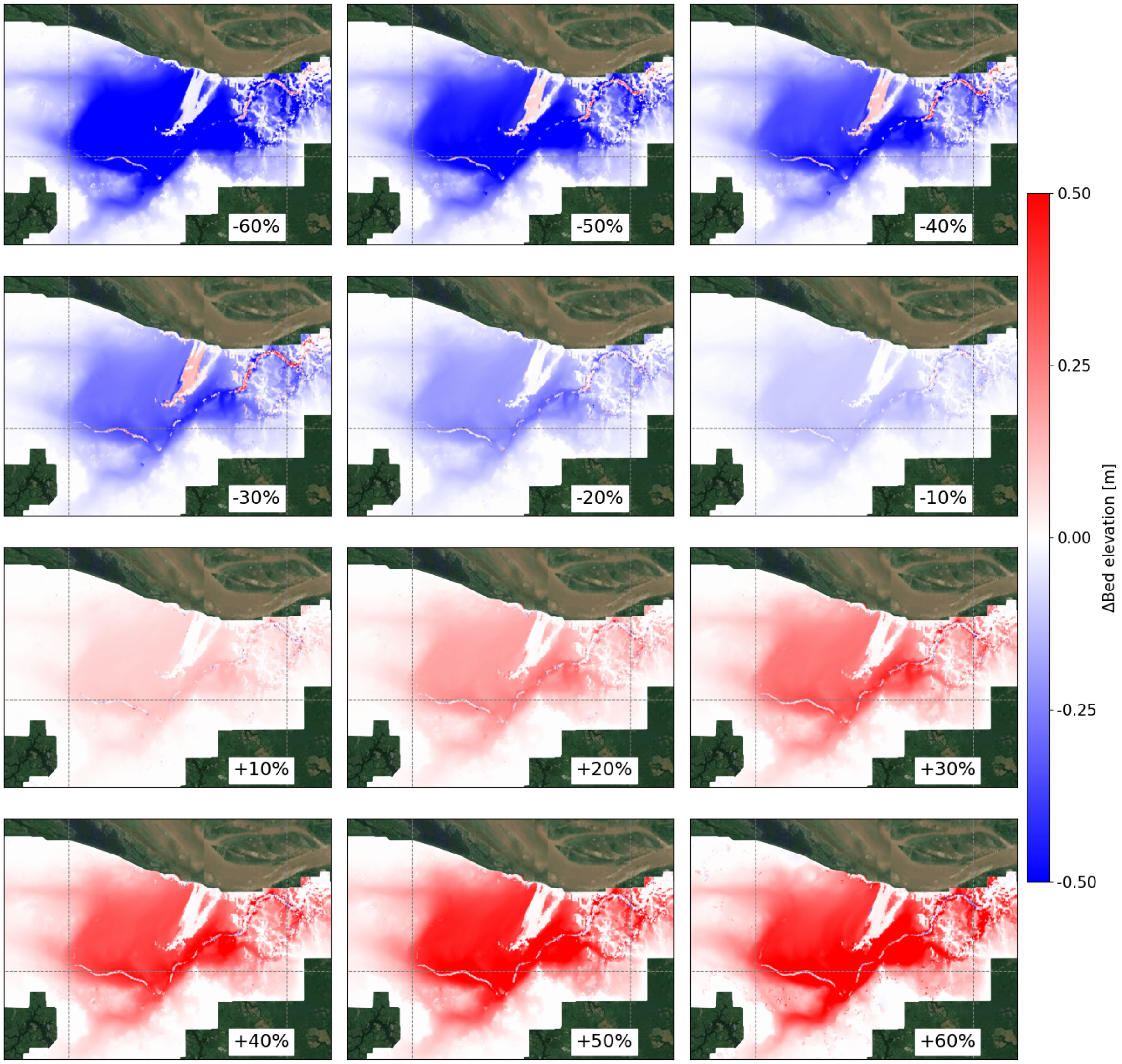

Figure S12 The difference of the total change of floodplain bed elevation from 2008 to 2016 between an experiment simulation and the baseline simulation (ΔBed elevation). The background maps are from Google Satellite Map (Map data ©2024 Google).

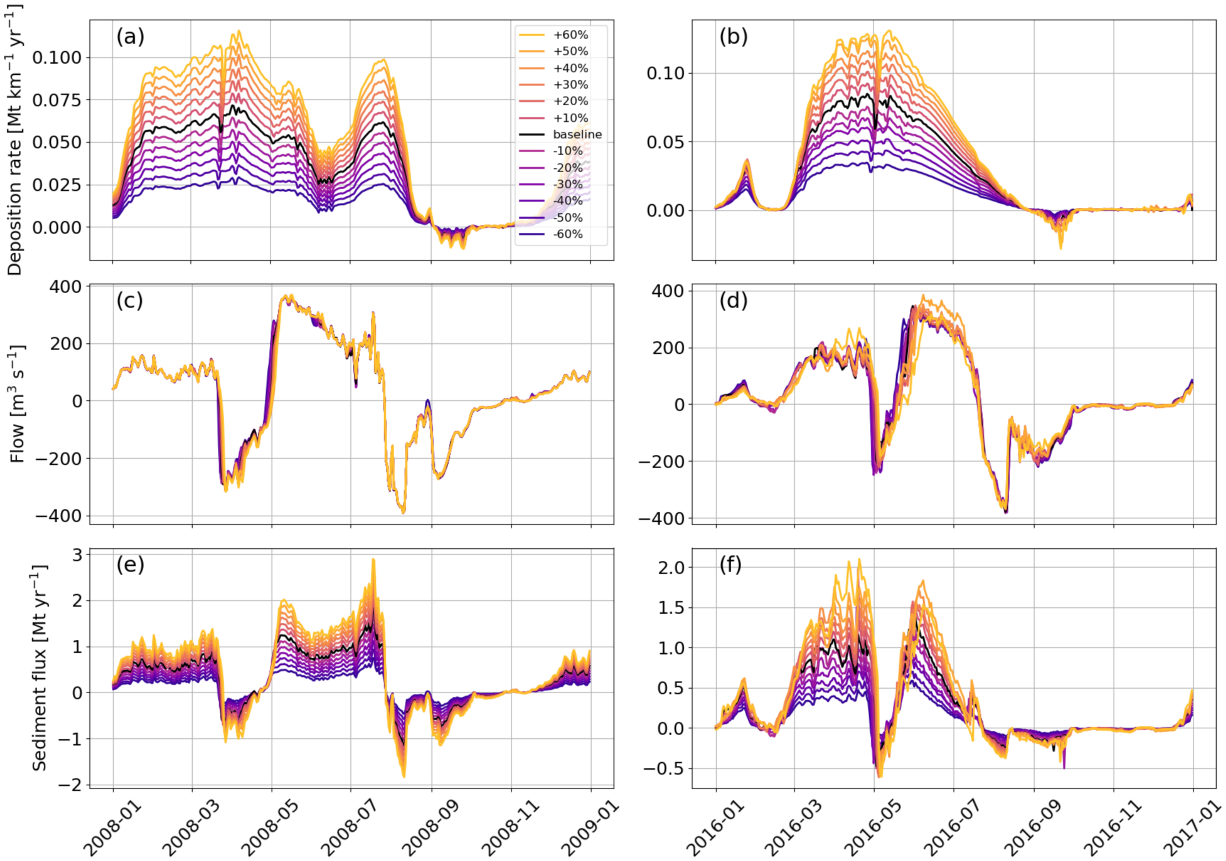

Figure S13 Time series of deposition rate in 2008 (a) and in 2016 (b) on different SSC perturbation levels. The corresponding exchanging rate of flow and sediment in 2008 (c and e) and in 2016 (d and f). Comparing the simulation results between 2008 and 2016, there is a clear difference in deposition rate and the exchange rate of flow and sediment. Even with only boundary SSC perturbed, alterations are observed in flow flux magnitude and timing at varying SSC levels in 2016 (d), impacting sediment flux (f). Source data are provided as a Source Data file.

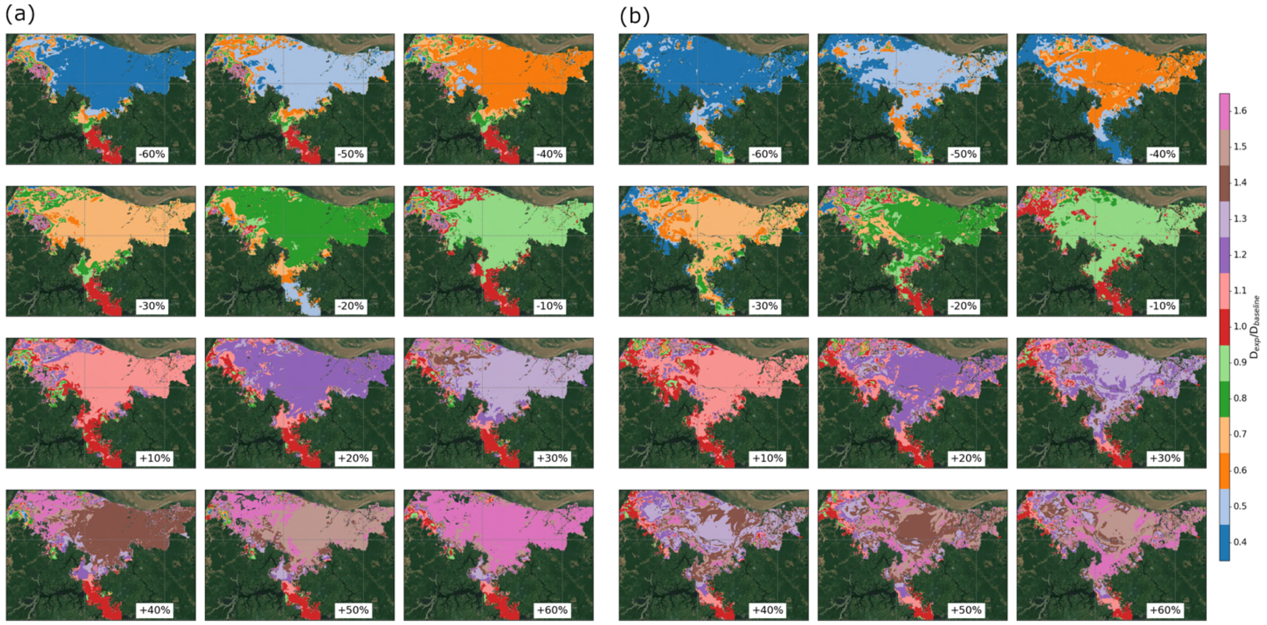

Figure S14 Spatial distribution of $D_{exp}/D_{baseline}$ in 2008 (a) and 2016 (b). In deeper regions near the floodplain's open boundary, $D_{exp}/D_{baseline}$ aligned with $SSC_{exp}/SSC_{baseline}$ in 2008 (a). However, in 2016, this consistency decreased, especially at higher SSC levels in (b), where the $D_{exp}/D_{baseline}$ typically fell below the corresponding $SSC_{exp}/SSC_{baseline}$. The background maps are from Google Satellite Map (Map data ©2024 Google).

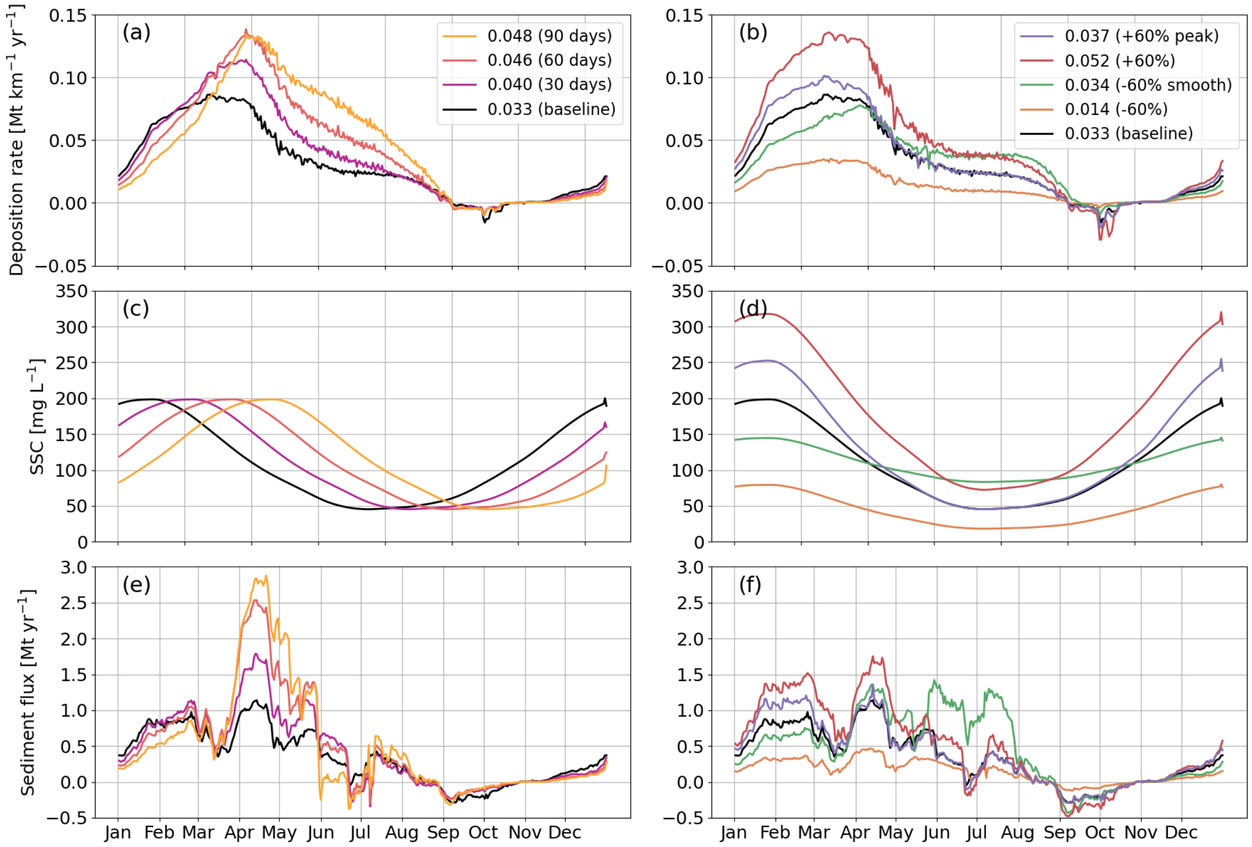

Figure S15 The daily averaged deposition rate (a), suspended sediment concentration (SSC) at the WLBCs (c) and sediment flux at the WLBCs (e) of the baseline simulation and the experiments that shift the SSC by 30 days, 60 days and 90 days towards the HW period. The daily averaged deposition rate (b), suspended sediment concentration (SSC) at the WLBCs (d) and sediment flux at the WLBCs (f) of the baseline simulation and the experiments that increases the SSC peak by 60% (+60% peak), increases the SSC by 60% (+60%), smooths the SSC variation by 60% (-60% smooth) and smooths the SSC by 60%. The number in front of each experiment name in the legends of (a) and (b) is the corresponding averaged deposition rate. These experiments show the substantial influence of intraannual variation in SSC and the dynamic response of sediment deposition rate. Notably, temporal shifts in SSC peaks led to significant changes in sediment influx, particularly when peaks coincide with the RW period, resulting in higher deposition rates, as shown in e. Conversely, smoothing the SSC peak by 60% did not reduce the deposition rate as much as reducing the entire SSC time series by the same percentage (b). This occurs because the smoothed SSC maintains higher values during both RW and HW compared to the baseline scenario. Moreover, we found that increasing SSC by 60\% during its peak days (d) does not lead to as high a deposition rate as increasing the entire time series by 60\% (b). This is because the SSC peak period slightly shifts among the simulation years, leading to a lower overall sediment flux and deposition rate (b and f). Source data are provided as a Source Data file.

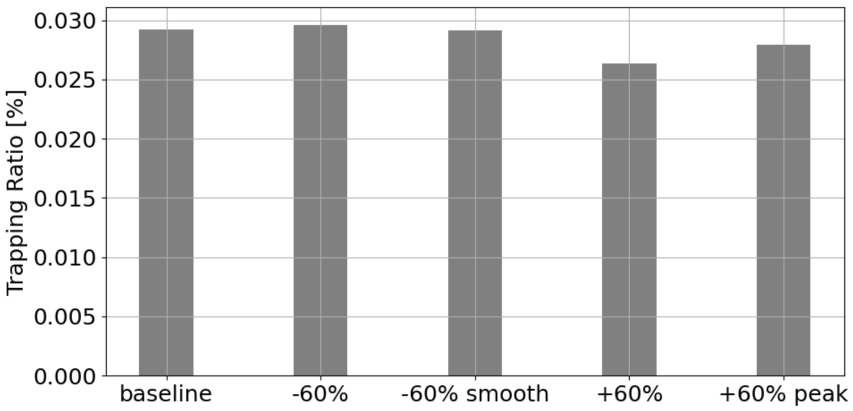

Figure S16 Annual averaged sediment trapping ratio in 2016 of the baseline simulation and the experiments that increases the SSC peak by 60% (+60% peak), increases the SSC by 60% (+60%), smooths the SSC variation by 60% (-60% smooth) and smooths the SSC by 60%. Source data are provided as a Source Data file.

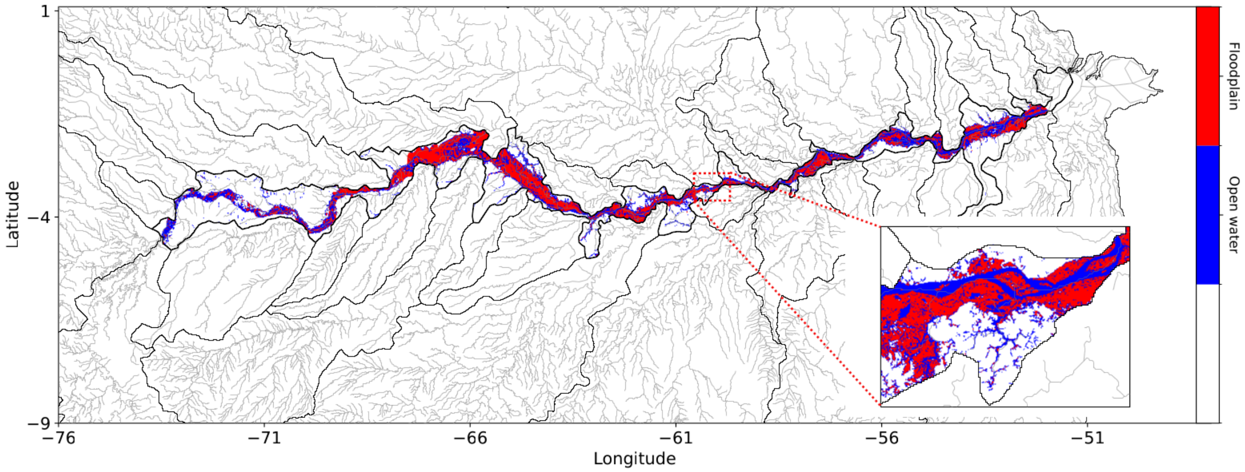

Figure S17 Floodplain and open water are directly delineated from the flooded wetland extent product at High Water Stage [1] within the drainage basin of the Amazon/Solimões River mainstem. The area of floodplains is 58,103 km2. The black lines represent the mainstem drainage area as well as major tributary subbasin. The gray lines are the stream network. The magnified view over the Janauacá floodplain. The shapefiles of subbasin boundaries and stream drainage directions are from [2].

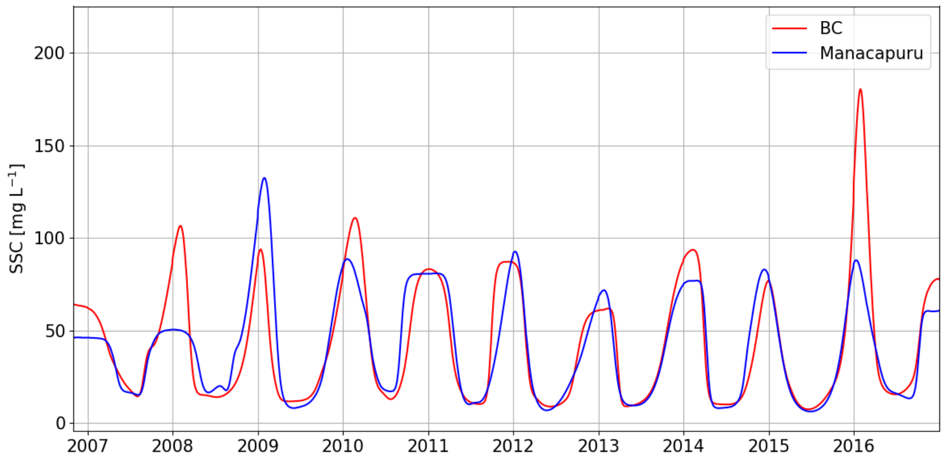

Figure S18 Time series of suspended sediment concentration (SSC) over the simulation period (2007~2016) at Manacapuru (blue) and at the Janauacá's open boundary (red). The SSC is extracted from the MODIS data processed in [3]. Source data are provided as a Source Data file.

Supplementary References

[1] Hess, L. L. et al. Wetlands of the lowland Amazon Basin: Extent, vegetative cover, and dual-season inundated area as mapped with JERS-1 synthetic aperture radar. Wetlands 35, 745–756 (2015).

[2] Mayorga, E., Logsdon, M. G., Ballester, M. V. R. & Richey, J. E. Estimating cell-to-cell land surface drainage paths from digital channel networks, with an application to the Amazon basin. J. Hydrol. 315, 167–182 (2005).

[3] Fassoni-Andrade, A. C. & de Paiva, R. C. D. Mapping spatial-temporal sediment dynamics of river-floodplains in the Amazon. *Remote sensing of environment* **221**, 94–107 (2019).
